# Arsenic trioxyde in the treatment of HTLV1 associated ATLL

**DOI:** 10.1186/1742-4690-8-S1-A59

**Published:** 2011-06-06

**Authors:** Felipe Suarez, Ambroise Marcais, David Ghez, Richard Delarue, Benedicte Deau-Fischer, Charbel Aoun, Flore Sicre de Fontbrune, Laurent Ysebaert, Vahid Asnafi, Daniele Canioni, Hugue deThe, Ali Bazarbachi, Olivier Hermine

**Affiliations:** 1 Hématologie Adultes, Groupe Hospitalier Necker - Enfants malades, Paris, France; 2 Hématologie Clinique, Institut Gustave Roussy, Villejuif, France; 3 Hématologie, CHU Purpan, Toulouse, France; 4 Hématologie Biologique, Groupe Hospitalier Necker - Enfants malades, Paris, France; 5 Anatomie Pathologique, Groupe Hospitalier Necker - Enfants malades, Paris, France; 6 CNRS Hôpital saint Louis, Paris , France; 7 Hematology - Internal Medicine, American University Hospital, Beyrout, Liban; 8 Hématologie Adultes et Cnrs Umr 8147, Groupe Hospitalier Necker - Enfants malades, Paris, France

## Background

The prognosis of Adult T-cell leukemia/lymphoma (ATLL) associated with HTLV1 is dismal. The response to conventional chemotherapy ranges between 20 and 70% and relaspe is constant. Median survival is 8 to 13 months. Chronic and smouldering ATLL have a longer survival, ranging from 18 to 72 months at 5 years. Interferon alpha (IFNa) and AZT combination therapy is effective in acute, chronic and smouldering ATLL, sometimes leading to complete response and has a better prognosis than conventional chemotherapy (5 year survival of 82% in acute and 100% in chronic and smouldering forms reaching a complete response). Patients with ATLL/lymphoma do not benefit from IFNa+AZT combination and despite initial response to chemotherapy, all patients eventually relapse. Arsenic trioxyde (AsO3) in combination with IFNa has in vitro activity with a negative regulation of the Tax oncoprotein and leads to apoptosis of HTLV1 transformed lymphocytes. A recent study has showed a benefit in chronic forms of ATLL.

## Patients and methods

11 patients with ATLL were treated with AsO3+IFN combination after a minimum of 1 line of treatment.

## Results

3 patients had ATLL/lymphoma, 3 chronic and 5 acute. All patients had recieved previous therapy with chemotherapy associated or not with IFNa/AZT combination. At initiation of AsO3, 4 patients were in complete response (3 lymphoma, 1 acute), 2 partial response (1 acute, 1 chronic) et 5 in progression (3 acute, 2 chronic). 10 patients recieved AsO3 during 3 to 8 weeks 1 patient progressed 3 days after starting AsO3. Tolerance was acceptable with peripheral neurolopathy (n = 4), hand and foot syndrome (n = 3) et drug eruption (n = 3 including 2 toxic epidermal necrolysis). 6 patients died, and all were progressing at time of AsO3 initiation. 5 patients survived : 3 lymphomas in complete response (25, 31 et 46 months follow-up), 1 acute in complete response (9 months follow-up) and 1 one chronic in partial response (39 months follow-up).

## Conclusion

AsO3 and IFNa combination has an acceptable tolerance profile and seems to be effective in ATLL in consolidation after response to a previous treatment, particularly in lymphoma and chronic forms.

